# 3D printed fluidics with embedded analytic functionality for automated reaction optimisation

**DOI:** 10.3762/bjoc.13.14

**Published:** 2017-01-18

**Authors:** Andrew J Capel, Andrew Wright, Matthew J Harding, George W Weaver, Yuqi Li, Russell A Harris, Steve Edmondson, Ruth D Goodridge, Steven D R Christie

**Affiliations:** 1 Department of Chemistry, Loughborough University, Loughborough, LE11 3TU, UK; 2 School of Mechanical Engineering, University of Leeds, Leeds, LS2 9JT, UK; 3 School of Materials, The University of Manchester, Manchester, M13 9PL, UK; 4 Faculty of Engineering, The University of Nottingham, Nottingham, NG7 2RD, UK

**Keywords:** 3D printing, inline reaction analysis, reaction optimisation, selective laser melting, stereolithography

## Abstract

Additive manufacturing or ‘3D printing’ is being developed as a novel manufacturing process for the production of bespoke micro- and milliscale fluidic devices. When coupled with online monitoring and optimisation software, this offers an advanced, customised method for performing automated chemical synthesis. This paper reports the use of two additive manufacturing processes, stereolithography and selective laser melting, to create multifunctional fluidic devices with embedded reaction monitoring capability. The selectively laser melted parts are the first published examples of multifunctional 3D printed metal fluidic devices. These devices allow high temperature and pressure chemistry to be performed in solvent systems destructive to the majority of devices manufactured via stereolithography, polymer jetting and fused deposition modelling processes previously utilised for this application. These devices were integrated with commercially available flow chemistry, chromatographic and spectroscopic analysis equipment, allowing automated online and inline optimisation of the reaction medium. This set-up allowed the optimisation of two reactions, a ketone functional group interconversion and a fused polycyclic heterocycle formation, via spectroscopic and chromatographic analysis.

## Introduction

Additive manufacturing (AM), or as it is widely known ‘3D printing’, is the internationally recognised term used to describe a wide range of manufacturing processes that can generate complex three-dimensional parts, often with geometries which would be extremely complex, or in some cases impossible to manufacture using more conventional subtractive manufacturing processes [1]. In AM, parts are built layer-by-layer, using processes such as material extrusion [2], material jetting [3], vat photopolymerisation [4], sheet lamination [5], powder bed fusion [6], binder jetting and direct energy deposition [7–8]. AM has gained widespread academic and industrial use for a diverse set of applications ranging from biological to aeronautical [9–10]. However, more recent research has demonstrated the benefits of using 3D printing to produce microfluidic devices using AM techniques such as stereolithography (SL) [11], polymer jetting and fused deposition modelling (FDM) [12–13]. There is therefore considerable interest in the optimisation of chemical systems using this type of multifunctional continuous flow reactor. Notable recent work in this area has been carried out by Cronin [14], Ley [15] and Jensen [16]. This research highlights the array of benefits that manufacturing fluidic devices via AM processes can bring, including the ability to produce multimaterial parts with complex microscale features and embedded functionality, allowing inline and online optimisation of a reaction medium.

This paper presents a range of printed chemical reactors produced via the selective laser melting (SLM) and SL manufacturing processes. SLM is a powder-based additive manufacturing technique which uses a high-power energy source, typically a laser, to selectively melt a powder bed into a single solid body [17]. SLM can manufacture parts in a range of chemically inert and thermally stable metals such as stainless steel [18], aluminium and titanium [19–20], and is therefore an attractive technique for a number of industrial applications. SLM is capable of producing parts at a layer thickness as low as 20 µm, and with part geometries of +/− 0.1 mm being achieved over smaller parts, however, even highly optimised SLM processes can still experience problems with balling, thermal cracking, unwanted surface roughness and difficulty with removing un-melted powder from smaller cavities [6]. SL utilises layer-by-layer photopolymerisation of a liquid resin bath to generate fully dense polymer parts [21]. Typically these resins are complex formulations based around a small selection of UV-curable acrylates, epoxies and urethanes [4], whose poor mechanical and chemical properties can limit the application of SL manufactured parts. However, well maintained machines are capable of reproducibly producing parts at a layer thickness as low as 25 µm, making SL one of the most accurate and reproducible AM processes [4]. Both SLM and SL are therefore attractive manufacturing techniques for the production of milliscale chemical reactors.

This research investigates how these two innovative processes can be used to produce milliscale chemical reactors with increased analytical functionality, by embedding spectroscopic viewing windows across the reaction path length allowing inline UV–vis spectroscopic analysis of the reaction medium. The research also highlights the design freedom associated with using AM processes, by designing custom reactor geometries which allow these devices to be integrated with existing laboratory flow and analysis equipment [22].

## Results and Discussion

Previous work within this research group has demonstrated the flexibility of AM for the production of milliscale chemical reactors, with complex internal geometries as well as parts with embedded spectroscopic capability [11,23]. In order to fully utilise this flexibility, parts were designed which could be integrated with existing flow and analytical instrumentation. An ideal choice for this application is high-performance liquid chromatography (HPLC). HPLC instrumentation is widely available in most modern chemistry laboratories, and is ideally suited for use in flow applications. Modern HPLC systems are typically equipped with a binary or quaternary pumping system (flow rates ≈0.01–10 mL/min), thermostatted heated compartments (temperatures ≈20–100 °C), multiport sampling valves, as well as separation, purification and UV–vis spectroscopic analysis capability. The HPLC system, parts were also integrated with a commercially available Uniqsis FlowSyn module providing pumping and heating apparatus, allowing inline spectroscopic reaction analysis via a portable UV–vis light source and detector. This type of spectroscopy is often used for inline reaction analysis due to its rapid data generation, however, it can often be difficult to interpret for complex multifunctional systems. On the other hand, chromatographic analysis methods produce much more concise spectra allowing quantitative data to be extrapolated, however, they often suffer from lengthy method times significantly decreasing the reaction throughput [24–25].

The HPLC equipment set-up, which varied between experiments, was based around a four module Agilent 1100 series, with two binary pumping modules, a thermostatted column compartment module, a variable wavelength diode array detector (DAD) compartment with a standard flow cell, as well as an external six-port sampling valve. Using this set-up allowed the flow medium to be pumped through a temperature controlled reactor, which using a sampling valve would allow the reaction medium to either be collected, injected onto the HPLC column for separation or passed directly through a diode array detector. The column would be flushed with the mobile phase by the secondary pump, whilst being independently heated by the same thermostatted compartment. By integrating this system with 3D printed fluidic devices, it would be possible to perform automated inline and online analysis of the reaction media, affording substantial control over reaction residence time, temperature, and reagent composition. However, in order to achieve this level of control it was necessary to design custom software: a series of intuitive ‘macro’ programs, which would allow the automated control of each module within the system. This required control over the Chemstation software that is the graphical user interface (GUI) for the Agilent HPLC system. This was achieved using MacroPad [26]. MacroPad is a software specifically designed for developing macros to control the Chemstation software. Through MacroPad, it is possible to access the Chemstation ‘registers’, which store all the input and output variables produced during the HPLC analysis. These registers allow control over variables such as reaction flow rates, temperature and pressure, as well as quantitative outputs such as spectroscopic and chromatographic data from any HPLC analysis undertaken. Using this software, it was possible to define the specific reaction and analysis conditions for each optimisation that was undertaken. More detailed descriptions of the function of the Chemstation macros and the SIMPLEX optimisation software are available in Supporting Information File 1. Both pieces of software allow user input, specifically defining the target variable to be optimized, e.g., absorption intensity or product peak area.

**Scheme 1:**
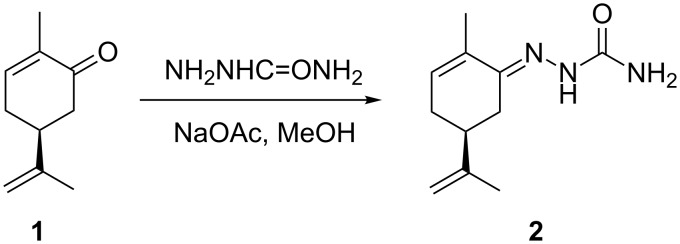
The reaction of (*R*)-(−)-carvone (**1**) with semicarbazide to form the corresponding semicarbazone **2**.

The large number of variables within the optimisation system and reactor design necessitated the generation of an idealised set of reaction conditions, allowing effective comparison of data sets. This reaction was the conversion of (*R*)-(−)-carvone (**1**) to its corresponding semicarbazone **2**, using semicarbazide and sodium acetate (Scheme 1). This reaction was selected because it would run smoothly at room temperature, and a mild solvent mixture such as methanol and water (MeOH/water) could be used with the less solvent-compatible parts. Differences between the UV–vis spectra of the starting material and the product can be used to follow the reaction optimization.

### Reactor design 1 (RD1)

**Figure 1:**
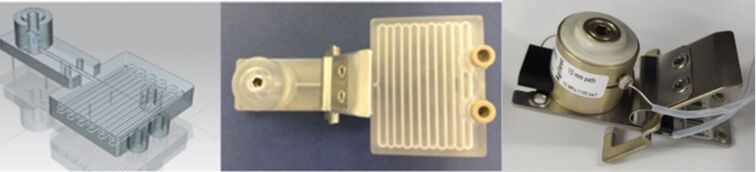
CAD model of SL reactor design RD1 (left), RD1 with attached sprung clip (centre), commercially available Agilent flow cell (right). External dimensions of RD1 are 123 (length) × 67 (width) × 42 mm (depth).

By mimicking the internal dimensions of the DAD compartment within an Agilent HPLC system, an inline spectroscopic flow cell could be realised (Figure 1). RD1 was therefore fabricated using a 3D Systems Viper si2 SL system from Accura 60 photoresin, with external geometries of 123 × 67 × 42 mm (volume ≈68 cm^3^), and a continuous cylindrical channel running throughout the part (channel diameter = 1.5 mm, channel length = 1600 mm, reaction volume = 2.8 mL). The external dimensions of the flow cell would match the internal dimensions of the DAD compartment, allowing the part to be held within by a commercially available sprung clip. The flow cell itself had a path length of 6 mm. One of the unique features of using AM to manufacture this type of part is that manufacturing costs are directly proportional to the volume of material used and not the complexity of the design. Despite the fact that the Accura 60 material has a high cost compared to conventional (non-SL) polymer materials, the material cost of RD1 is only around £17, making the SL process reasonably priced in comparison to other manufacturing processes.

**Figure 2:**
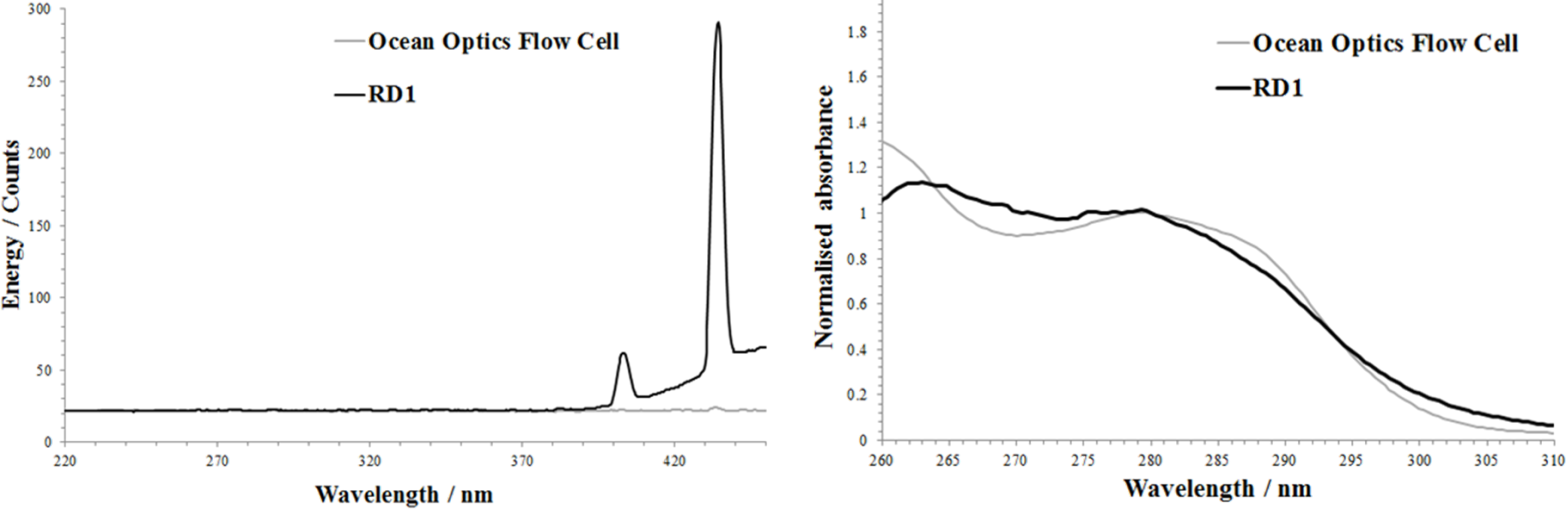
Energy versus wavelength spectra comparing the amount of stray light being picked up by the detector using both RD1 and a commercial flow cell (left), normalised absorption spectra of a benzaldehdye solution passing through both RD1 and a commercial flow cell (right).

The functionality of RD1 was determined via the use of an Ocean optics DH2000 light source (400 micron diameter illumination fibre, 600 micron collection fibre) and an Ocean Optics S2000 variable wavelength detector [27]. It was possible to determine the amount of stray light (predominantly from fluorescent strip lighting within the laboratory) being picked up by the detector when the light source was inactive (Figure 2). This demonstrated that due to the transparency of the Accura 60 resin to visible light, even though the part would be housed inside a dark chamber, ideally the detection wavelengths for this material should be kept below 400 nm. To confirm that the part functions correctly, a benzaldehyde solution (2 mmol in methanol), was flowed through RD1 with the resulting spectrum being compared to that achieved through the Ocean Optics flow cell. Having normalised the data it was clear that the two spectra were very similar above wavelengths of around 260 nm.

**Figure 3:**
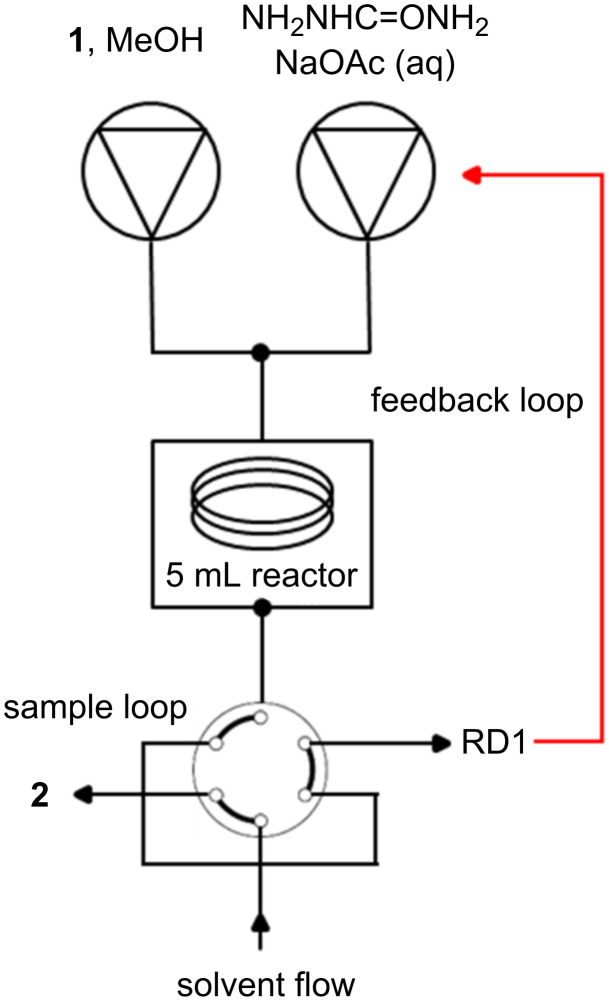
Reactor set-up for carvone optimisation using RD1 as an inline spectroscopic flow cell. Reagents were pumped using an Agilent 1100 series HPLC pumping module. A Uniqsis FlowSyn was used to heat and cool the 5 mL stainless steel coil reactor. The flow passed onto a stand-alone six-port valve, whereby samples were either passed into a collection vial or passed through RD1 which sat within the DAD compartment of the same Agilent 1100 series HPLC.

RD1 was therefore tested using the carvone functional group interconversion previously outlined (Scheme 1) and would be fully automated, using the spectroscopic data generated from the inline flow cell as the controlling output that would run the Chemstation control macros and optimisation software. The software was set to optimise for maximum UV–vis absorbance due to the semicarbazone by automatically varying both temperature and flow rate. For this optimisation an Agilent 1100 series binary pumping module was used to pump the two reagent flows, which passed through a 5 mL stainless steel coil reactor. This reactor was attached to a heating mandrel, and heated using the temperature controlled heating module of a Uniqsis FlowSyn. The flow would then pass into a six-port valve, allowing it to be redirected into either a collection vial, or pass through RD1 for spectroscopic data collection (Figure 3 and Table 1).

**Table 1:** Conditions and limits for the optimisation used in tandem with RD1. Ketone **1** concentration 0.40 mmol/L, semicarbazide concentration 1.20 mmol/L.

Optimisation variable	Value

flow rate range	0.2–1 mL/min
temperature range	25–80 °C
SIMPLEX temperature variation	5 °C
SIMPLEX flow rate variation	0.1 mL/min
maximum data points	30

**Figure 4:**
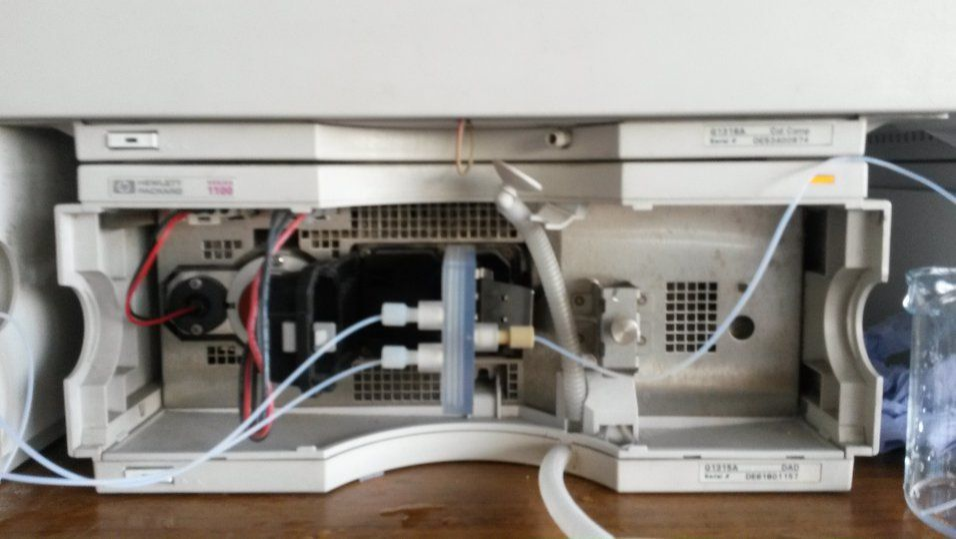
RD1 held in place within the DAD compartment of an Agilent 1100 HPLC.

The analysis macro used during this specific optimisation would monitor the intensity of absorption at a single predetermined wavelength (275 nm). At this wavelength the carvone starting material has very low absorbance, whereas the semicarbazone product has significant absorbance. The increase in intensity of absorbance at this value could therefore be attributed to the presence of an increased concentration of the reaction product. Prior to each new set of experimental conditions, the flow cell would be flushed with a MeOH/water mix (1:1 ratio), allowing the detector to establish a new baseline. Figure 4 shows the reactor held in place in the HPLC compartment.

**Figure 5:**
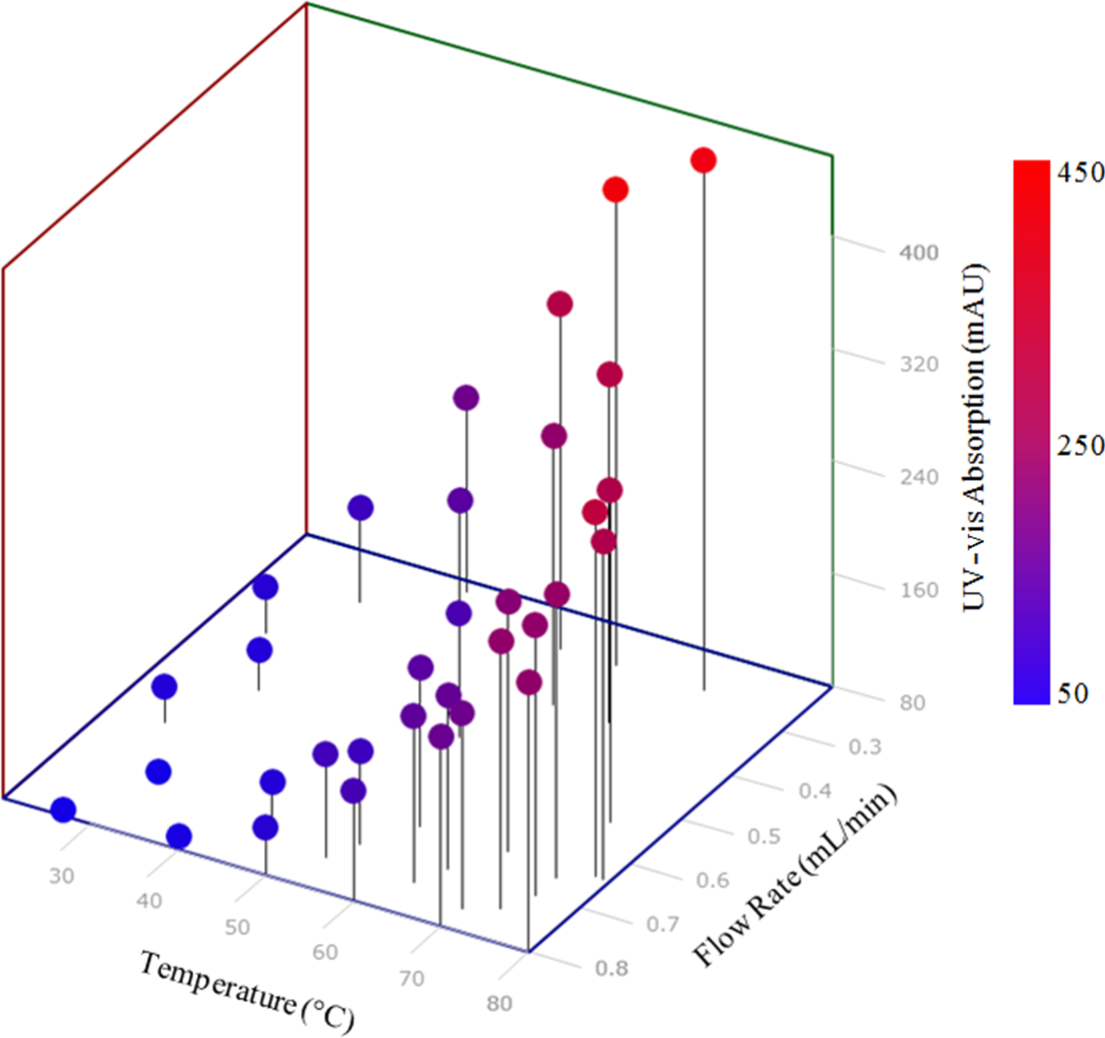
Optimisation plot for the SIMPLEX optimisation of semicarbazone **2**. Optimum reaction conditions within the specified system were found to a flow rate of 0.27 mL/min and a temperature of 69 °C.

The optimisation was run over the period of approximately 8 h, generating 30 data points within the allowable temperature and flow rate range (Figure 5). Successive points were automatically selected by the SIMPLEX algorithm using previous results, in order to find the optimum conditions. This produced the optimal data point as being 69 °C and 0.27 mL/min (Figure 5). This initial optimisation methodology was able to quickly identify the trend towards higher yield with higher temperatures and lower flow rates. This type of analysis is ideal for fast data generation. Indeed, with this type of analysis the biggest delay within the system was the wait for the heating and cooling of the reactor between analysis points. However, this analysis method did have a number of features which could be improved upon with future design alterations. The use of both a FlowSyn and HPLC system made it complex to co-ordinate both pieces of instrumentation. Also the reaction could not be carried out at uniform temperature throughout due to poor thermal conductivity and stability of the Accura material that RD1 was manufactured with. It was hypothesised however, that both of these features could be overcome by manufacturing reactors via the SLM process, allowing the parts to be manufactured from thermally conductive and thermally stable metals that could be designed to retrofit to any off-the-shelf heating device (see RD2 below).

### Reactor design 2 (RD2)

**Figure 6:**
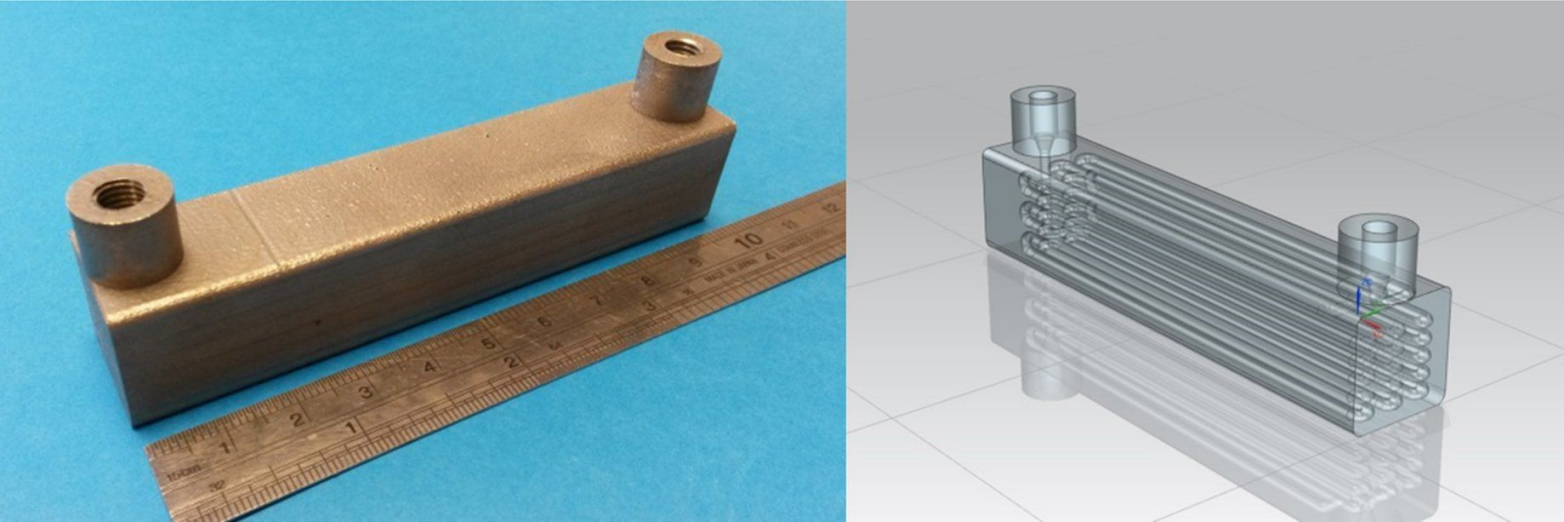
SLM reactor RD2 (left), CAD model of RD2 (right). External dimensions of RD2 are 100 (length) × 20 (width) × 20 mm (depth).

Agilent HPLC systems are equipped with two programmable temperature controlled column compartments, which allow temperatures to be independently heated up to 100 °C and simultaneously selected by the user. This will allow a bespoke chemical reactor to be placed into one of these compartments, whilst allowing the inline separation and analysis of reaction products downstream of the device. This set-up allows the temperature controlled reaction, purification, analysis and optimisation of a reaction medium all within a single piece of common laboratory equipment. RD2 was therefore designed to match the internal dimensions of the heated column compartment of an Agilent 1100 series HPLC system (Figure 6). The part was fabricated using a Renishaw AM 250 system from Ti-6Al-4V alloy, with external geometries of 100 × 20 × 20 mm (volume = 31.6 cm^3^) and a continuous cylindrical channel running throughout (channel diameter = 2 mm, channel length = 3200 mm, theoretical reaction volume = 10 mL). The titanium alloy used to manufacture the part is thermally stable across a substantial temperature range, and chemically compatible with a wide range of organic solvents and reagents, making it ideally suited to continuous flow chemistry.

**Figure 7:**
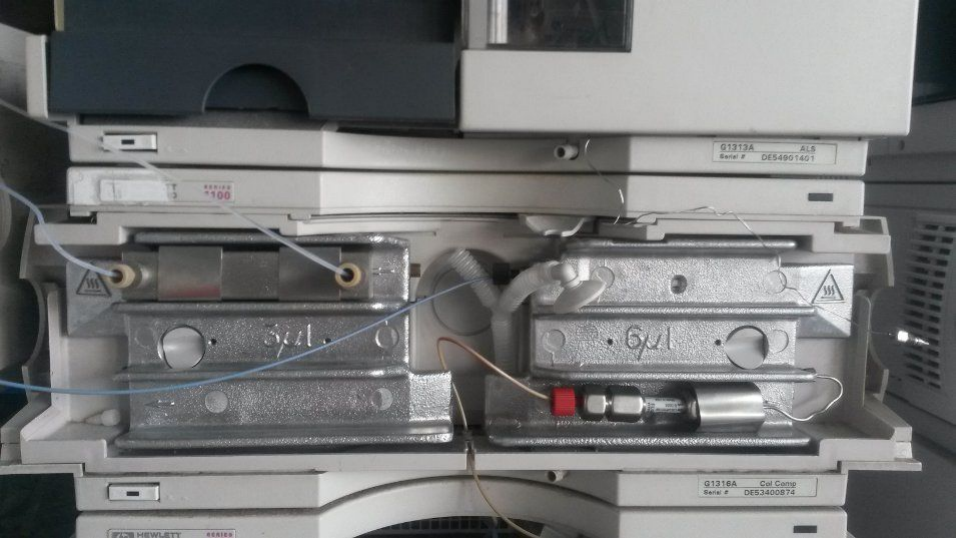
RD2 held in place within the thermostatted Agilent 1100 series column department.

The part was again tested using the semicarbazide preparation previously outlined (Scheme 1), and automated through the Chemstation software. For this optimisation an 1100 series binary pump module was used to pump the two reagent flows directly through RD2. The part was placed into the HPLC column compartment (Figure 7), and heated using the temperature control settings within the Chemstation software. The flow would then pass into a six port sampling valve, allowing the material to pass into either a collection vial, or be injected directly onto the HPLC column for purification and further analysis. To verify the actual temperature versus the set temperature, we flowed a methanol/water mix through the set-up and measured the temperature at the reactor exit. We did see an offset of around 5 °C for every set increase of 20 °C. Whilst there is predictability in this, this confirms that accurate reaction temperature measurement would be desirable in future design. For further details regarding the experimental set-up see Supporting Information File 1.

**Figure 8:**
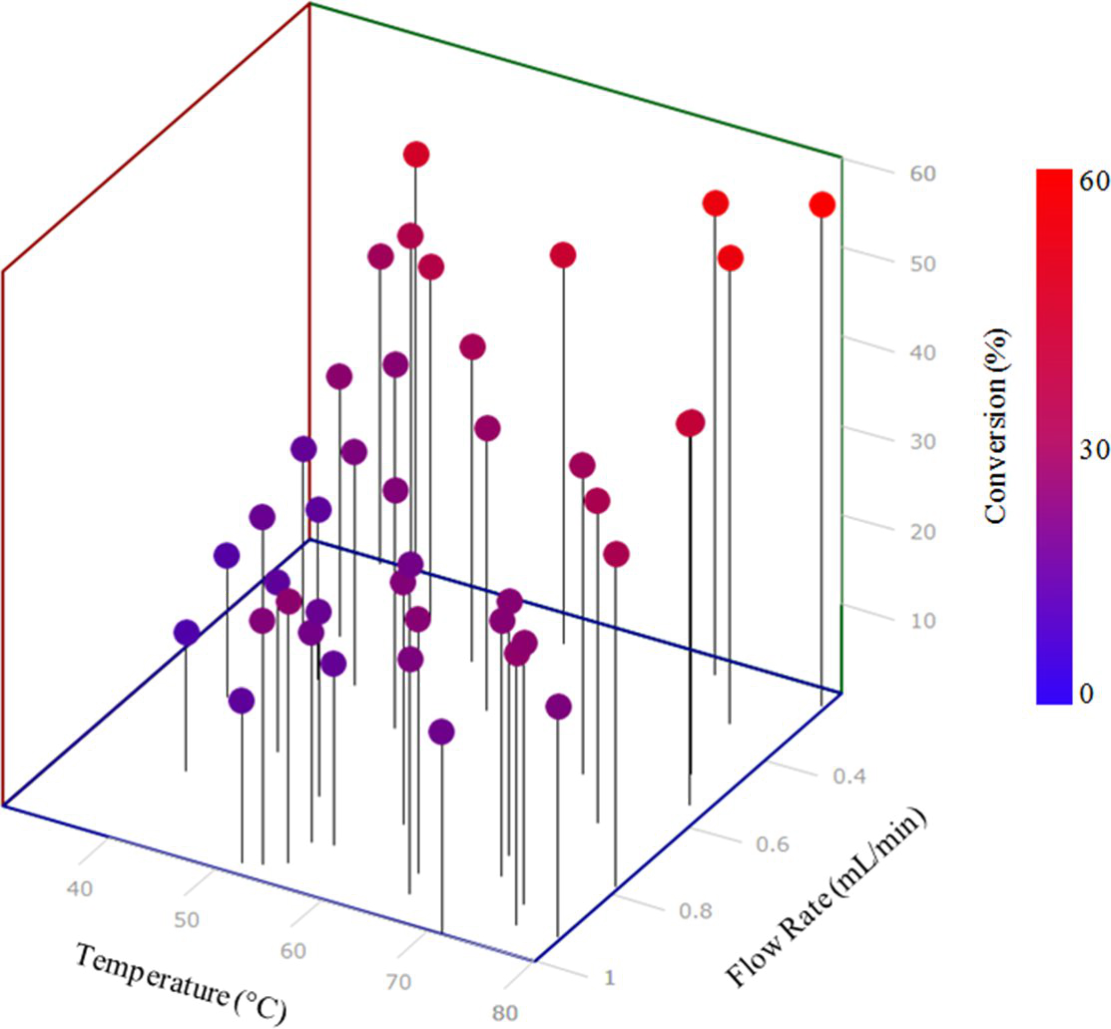
Optimisation plot for the SIMPLEX optimisation of semicarbazone **1**. Optimum reaction conditions were found to be a flow rate of 0.24 mL/min and a temperature of 79.6 °C.

The specific macro used during this optimisation was set up to calculate the peak area for both the carvone starting material, as well as the semicarbazone product. The percentage conversion of the starting material was then outputted as a single value. The optimisation was run over the period of around 24 hours, generating 40 data points within the allowable temperature and flow rate range (Figure 8). This produced the optimal reaction conditions as being 79.6 °C and 0.24 mL/min, which had a conversion of 56%. Again the system was able to identify the general trend towards higher yields at lower flow rates and higher temperatures. However, switching from spectroscopic to chromatographic analysis caused a significant increase in the amount of time required to complete the optimisation, with each data point taking around 35 minutes to generate. However, the system did produce much more easily-quantifiable spectra resulting in a significant improvement in the reliability and accuracy of the data generated.

**Scheme 2:**
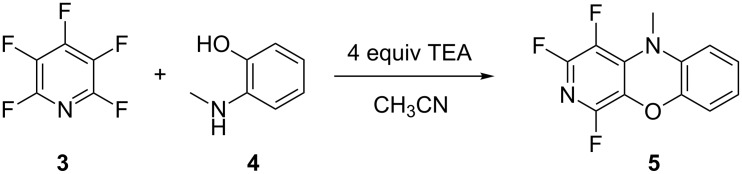
The reaction of pentafluoropyridine (**3**) with 2-(methylamino)phenol (**4**) to form the corresponding fused polycyclic heterocycle **5**.

The thermal and chemical stability of the Ti-6Al-4V alloy used to manufacture RD2 opened up a much wider range of potential chemical syntheses possible using this device. It was hypothesised that integrating RD2 with a commercially available FlowSyn module would allow a much larger chemical space to be analysed (<200 °C). The formation of a fused polycyclic heterocycle **5** (Scheme 2), from pentafluoropyridine (**3**) and 2-(methylamino)phenol (**4**), was chosen as this would generate a more complex optimisation set with two starting materials, the reaction product as well as any potential reaction intermediates and unwanted side products. The reaction would also require elevated temperatures as well as a solvent system which would have proved destructive to the Accura resin used to manufacture RD1. These types of fused polycyclic heterocycles are of significant interest, as they have been shown to have significant antitrypanosomal activities against *Trypanosoma brucei rhodesiense,* with low or no toxicity towards mammalian cells [28], thus testing the system against a real research problem.

The reaction set-up for this optimisation consisted of RD2 being held into place on the chip heater of a FlowSyn system by a metal clip. The system was allowed to reach temperature with solvent pumping throughout the system, before switching to a reagent flow. The product from each optimisation point was collected and analysed via UV–vis spectroscopy at a wavelength of 330 nm. For further details regarding the experimental set-up see Supporting Information File 1.

**Figure 9:**
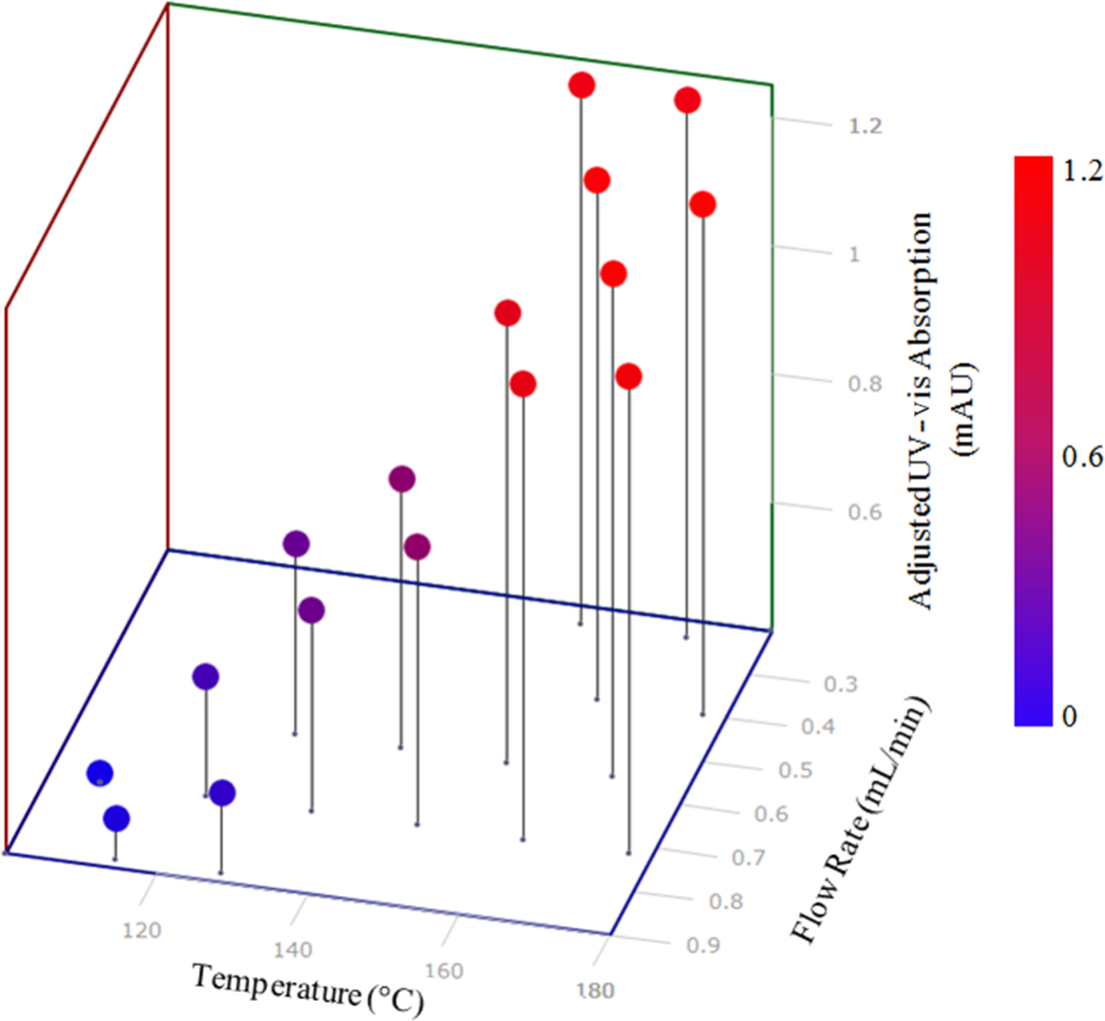
Optimisation plot for the SIMPLEX optimisation of the fused polycyclic heterocycle **5**. Two optimal data points at 0.24 mL/min and 156 °C, and 0.24 mL/min and 170 °C were found.

The optimisation generated two optimal data points at 0.24 mL/min and 156 °C, and 0.24 mL/min and 170 °C, respectively (Figure 9). Despite a 12-fold increase in reaction conversion over the course of the optimisation, the optimum data point generated correlated to only around 23.4% conversion. This output does perhaps suggest that at a lower flow rate, or higher residence time, a more optimal set of reaction conditions could be realised. Limitations of the current pumping system used above made it impractical to drop to a lower flow rate, however, the inherent benefit of AM processes is that a new reactor design with a larger internal reaction volume can be realised within a short time period. In this manner AM affords the opportunity to design and develop reactor geometries, specifically tailored to the individual needs of the reaction in use, be that in terms of reactor dimensions or specific analysis sites located throughout the port, in a highly cost and time efficient manner. If coupled with HPLC purification of target compounds, it offers a rapid method for generation of quantitites of compounds for further testing.

### Reactor design 3 (RD3)

**Figure 10:**
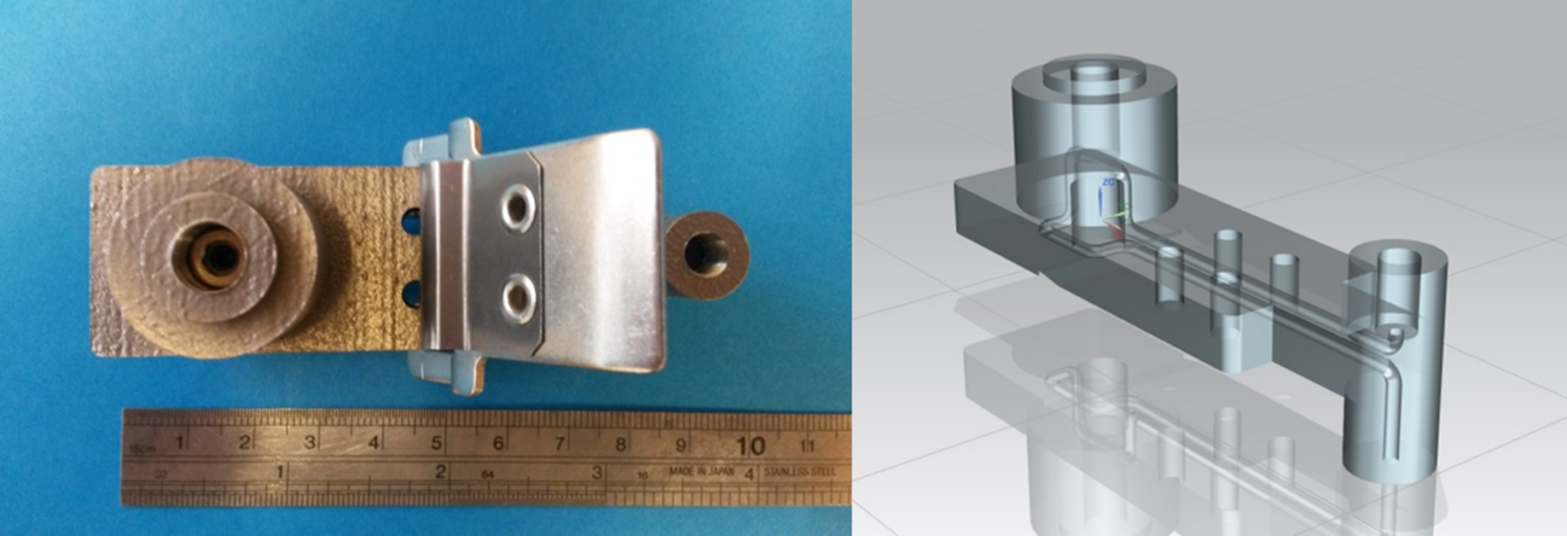
SLM reactor design RD3 (left), CAD model of RD3 (right). External dimensions of RD3 are 89 (length) × 27 (width) × 38 mm (depth).

Having previously demonstrated that it was possible to manufacture a flow cell with in-build windows from polymer via the SL process (RD1), it was logical to produce a similar part from metal. This would allow high and low-temperature reactions to be undertaken, in a much larger range of chemical reagent and solvents. RD3 was again produced using a Renishaw AM 250 system from Ti-6Al-4V alloy, with external geometries of 89 × 27 × 38 mm (volume = 24.6 cm^3^) and a continuous cylindrical channel running throughout (channel diameter = 2 mm, channel length = 190 mm, reaction volume = 0.6 mL) (Figure 10). Like RD1, the external dimensions of the flow cell would match the internal dimensions of the DAD compartment, whereby a flow cell of path length 2 mm would sit approximately half way along the flow path . For further details regarding the experimental set-up see Supporting Information File 1.

**Figure 11:**
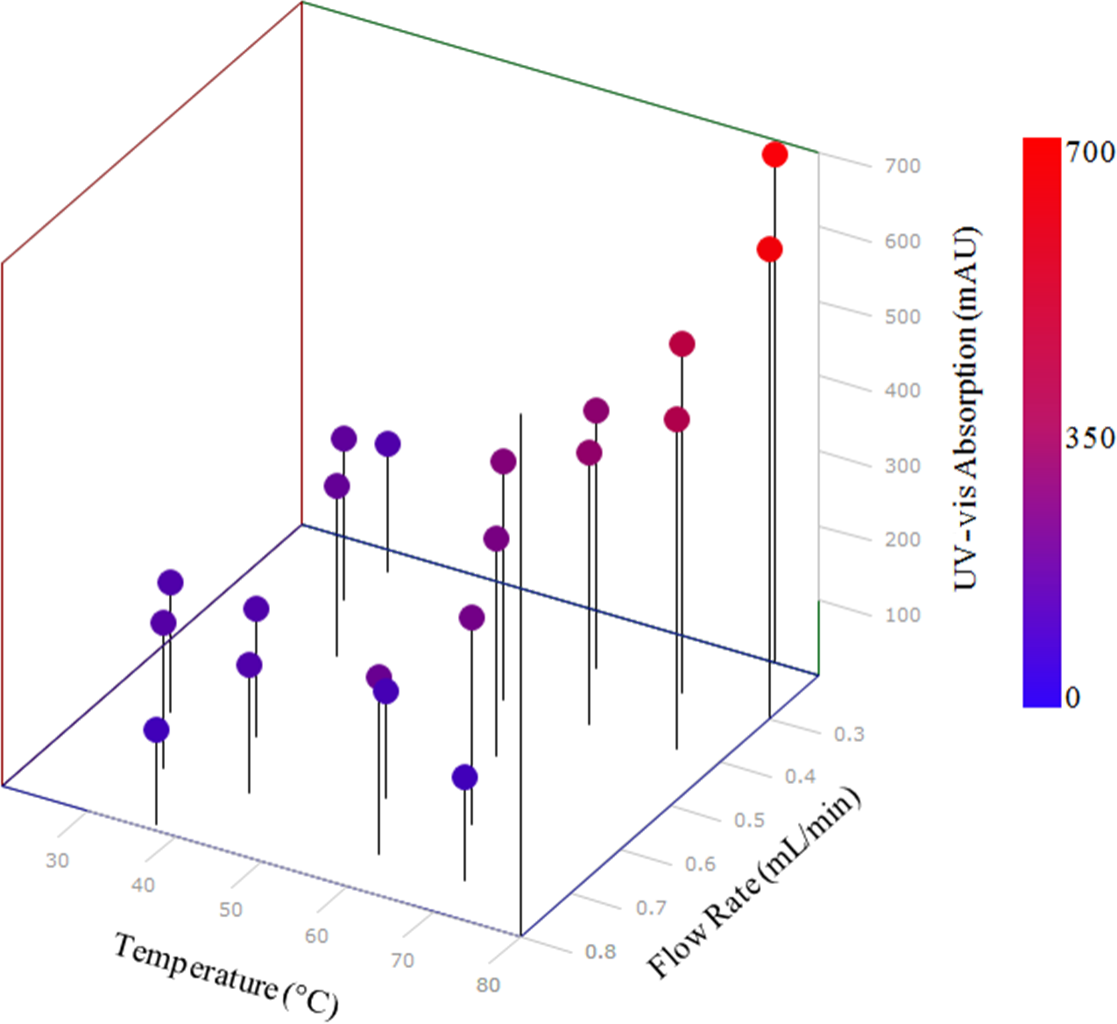
Optimisation plot for the SIMPLEX optimisation of semicarbazone **2**. Optimum reaction conditions were found to be a flow rate of 0.2 mL/min and a temperature of 75 °C.

Again the part was tested using the model semicarbazone reaction and the same optimisation set-up used during the testing of RD1. However, the 5 mL stainless steel reaction coil previously used was replaced by RD3, which sat in the temperature controlled column compartment of the HPLC. This increased the total internal reaction volume to around 10.3 mL. This set-up meant that the entire reaction, analysis and optimisation would be performed within a single HPLC system, using only AM parts. The optimisation was run over the period of about 6 hours, generating 20 data points within the allowable temperature and flow rate range. This produced the optimal data point as being 75 °C and 0.2 mL/min (Figure 11). Both RD2 and RD3 have demonstrated the immense potential of AM processes to not only manufacture bespoke and customisable geometries which can be integrated with existing laboratory equipment, but also to manufacture functional chemical and thermally compatible reactors with embedded functionality.

## Conclusion

AM has been shown to be a highly versatile manufacturing process for the production of multifunctional bespoke flow reactors. This allows conceptual parts to be realised within a short time period, and consequently a rapid optimisation of the designed geometry can be achieved. The customisable nature of the AM process allowed the generation of a selection of custom built metal and polymer parts. These parts were designed so that they could be integrated with existing pieces of flow and analysis instrumentation, as well as housing analytical functionality in the form of spectroscopic windows. By integrating this type of custom-made device with a piece of intuitive software, it was possible to develop a fully automated flow system capable of generating a significant amount of data at discrete locations within the flow system. There is therefore significant future research scope in this area where additive manufacturing offers the ability to embed analytical technology in reactors in innovative ways.

## Supporting Information

File 1 General considerations, macros and experimental data.

## References

[1] Wong K V, Hernandez A (2012). ISRN Mech Eng.

[2] Masood S H (1996). Rapid Prototyp J.

[3] Shallan A I, Smejkal P, Corban M, Guijt R M, Breadmore M C (2014). Anal Chem.

[4] Melchels F P W, Feijen J, Grijpma D W (2010). Biomaterials.

[5] Friel R J, Harris R A (2013). Procedia CIRP.

[6] Kruth J P, Froyen L, Van Vaerenbergh J, Mercelis P, Rombouts M, Lauwers B (2004). J Mater Process Technol.

[7] Bai Y, Williams C B (2015). Rapid Prototyp J.

[8] Carroll B E, Palmer T A, Beese A M (2015). Acta Mater.

[9] Zein I, Hutmacher D W, Tan K C, Teoh S H (2002). Biomaterials.

[10] Brandl E, Baufeld B, Leyens C, Gault R (2010). Phys Procedia.

[11] Monaghan T, Harding M J, Harris R A, Friel R J, Christie S D R (2016). Lab Chip.

[12] Anderson K B, Lockwood S Y, Martin R S, Spence D M (2013). Anal Chem.

[13] Capel A J, Edmondson S, Christie S D R, Goodridge R D, Bibb R J, Thurstans M (2013). Lab Chip.

[14] Tsuda S, Jaffery H, Doran D, Hezwani M, Robbins P J, Yoshida M, Cronin L (2015). PLoS One.

[15] Fitzpatrick D E, Battilocchio C, Ley S V (2016). Org Process Res Dev.

[16] Moore J S, Smith C D, Jensen K F (2016). React Chem Eng.

[17] Yadroitsev I, Smurov I (2011). Phys Procedia.

[18] Rombouts M, Kruth J P, Froyen L, Mercelis P (2006). CIRP Ann.

[19] Louvis E, Fox P, Sutcliffe C J (2011). J Mater Process Technol.

[20] Thijs L, Verhaeghe F, Craeghs T, Van Humbeeck J, Kruth J-P (2010). Acta Mater.

[21] Sager B, Rosen D W, Shilling M, Kurfess T R (2003). Experimental Studies in Stereolithography Resolution. Proceedings of the Annual International Solid Freeform Fabrication Symposium.

[22] Kitson P J, Glatzel S, Chen W, Lin C-G, Song Y-F, Cronin L (2016). Nat Protoc.

[23] Monaghan T, Capel A J, Christie S D, Harris R A, Friel R J (2015). Composites, Part A.

[24] Holmes N, Akien G R, Savage R J D, Stanetty C, Baxendale I R, Blacker A J, Taylor B A, Woodward R L, Meadows R E, Bourne R A (2016). React Chem Eng.

[25] Sans V, Cronin L (2016). Chem Soc Rev.

[26] (2015). MacroPad.

[27] (2015). OceanOptics.

[28] Brown-Barber C J (2010).

