# Supplementary material for: 3D printed fluidics with embedded analytic functionality for automated reaction optimisation
Source: Beilstein J Org Chem. 2017 Jan 18;13:111–9. doi: 10.3762/bjoc.13.14 (PMC5302008; doi:10.3762/bjoc.13.14)
Supplement: File 1 — General considerations, macros and experimental data. [file Beilstein_J_Org_Chem-13-111-s001.pdf]

Supporting Information

for

3D printed fluidics with embedded analytic functionality for automated reaction optimisation

Andrew J. Capel[1], Andrew Wright[1], Matthew J. Harding[1], George W. Weaver[1], Yuqi Li[1], Russell A. Harris[2], Steve Edmondson[3], Ruth D. Goodridge[4] and Steven D. R. Christie*[1]

Address: [1]Department of Chemistry, Loughborough University, Loughborough, LE11 3TU, UK, [2]School of Mechanical Engineering, University of Leeds, Leeds, LS2 9JT, UK, [3]School of Materials, The University of Manchester, Manchester, M13 9PL, UK and [4]Faculty of Engineering, The University of Nottingham, Nottingham, NG7 2RD, UK

Email Address: Steven D. R. Christie* - S.D.Christie@lboro.ac.uk

* Corresponding author

General considerations, macros and experimental data

Table of Contents

1. General considerations……………………………………………………………………….……….....S2

1.1 Software……………..…………………………………………………………………S2

1.2 Additive manufacturing equipment……………………………………………………….S2

1.3 Flow chemistry and analysis instrumentation ……………………………………………...S3

2. Macros……………………………………………………………………………………………….....S3

2.1 Chemstation macros………………………………………………………………......S3

2.2 MacroPad macros…………………………………………………………………….....S5

3 Experimental……………………………………………………………………………………………..S5

3.1 General procedure for the formation of carvone semicarbazide. ……………………………..S5

3.2 General procedure for the formation of the fused polycyclic heterocylcle ……………………..S5

3.3 Experimental details for RD1……………………………………………………………..…...S6

3.4 Experimental details for RD2………………………………………………………………….S7

3.5 Experimental details for RD3………………………………………………………………….S9

3.6. Reactor temperature verification……………………………………………………………S9

1. General considerations

1.1 Software

CAD drawings were produced using the commercially available software NX 7.5 (Siemens Industry Software), and converted to .STL file format for visualisation in MiniMagics 2.0 (Materialise).

Macros were developed using a trial version of Macropad and Microsoft Visual Basics.

1.2 Additive manufacturing equipment

Selective Laser Melting (SLM) – SLM parts were produced using a Renishaw AM250 system from Ti-6Al-4V alloy. Typical machine build settings are outlined in the following table.

Table S1: SLM Build parameters.

**Outer Shell Build Parameters**	**Value**
Build Style	Contour
Contours	1330
Hatches	167383
Volume Border Build Parameters	Value
Distance	52.6 m
Exposure Time	14.6 h
Speed	1 mm/s
Power Output	140 W
Focus Offset	11 mm
Point Distance	20 µm
Point Exposure Time	65 µs
Volume Area Build Parameters	Value
Distance	1238.6 m
Exposure Time	344.1 h
Speed	1 mm/s
Power Output	200 W
Focus Offset	-12.5 mm
Point Distance	45 µm
Point Exposure Time	190 µs
Volume Area Build Parameters	Value
Distance	51.9 m
Exposure Time	14.4 h
Speed	1 mm/s
Power Output	200 W
Focus Offset	11 mm
Point Distance	65 µm
Point Exposure Time	225 µs

Stereolithography Apparatus (SLA) – SL parts were produced using a 3D Systems Viper si2 SLA system from Accura® 60. Typical machine build settings are outlined in the following table.

Table S2: SLA build parameters.

**Setting**	**Value**
Laser Power	18 mW
Border speed	10.4 units/s
Hatch speed	50.7 units/s
Fill speed	27.1 units/s
Material	Accura 60
Dip Distance	0

Pre Drip	0
Z Wait	0
Sweeps	1
Sweep Velocity	1.9685 units/s
Preferred Blade Gap	0.0120 in
Layer Thickness	0.1 mm
Blade gap %	400
Blade Velocity	50 units/s

1.3 Flow chemistry and analysis instrumentation

Pumping instrumentation – Flow was achieved using two pumping systems; an Agilent 1100 series pumping module as well as a Uniqsis FlowSyn. External tubing used in the syntheses was typically 1 mm I.D stainless steel.

Heating instrumentation – Reactions were heated using either an Agilent 1100 series heating module or a Uniqsis Flowsyn.

Analysis instrumentation – All HPLC analysis was conducted using an Agilent 1100 series LC system. This system consisted of two binary pumping modules, an autosampler, heated column compartment, and DAD. The column used was an Agilent Zorbax Eclipse Plus C18, 4.6 × 50 mm, 3.5 μm. Details of individual LC runs are given in the results and discussion section of the main paper. UV–vis spectroscopy was carried out using an Ocean optics DH2000 light source (400 micron diameter illumination fibre, 600 micron collection fibre) and an Ocean Optics S2000 variable wavelength detector.

2. Macros

2.1 Chemstation macros

The following is a description of the function of the macros that were written, and how they operated within the Chemstation software.

Having opened the Chemstation software, the user must load the optimisation macros using the Chemstation dialog input box. The user must then create a template method, which defines the initial reaction and analysis conditions for the pumping modules, column compartment, DAD compartment, as well as the external 6 port valve. The 6 port valve is programmed to inject a slug of reaction medium onto the column at the beginning of each new LC method. This template can be saved and re-opened for future run sequences. This method will define the reaction and analysis conditions which each of the optimisation runs will be based around. For the "Create Optimisation Set" tab the user must generate a .txt file containing the set of reaction conditions which are to be run.
Upon loading of the Chemstation macros, a new drop down menu called "Reaction Monitoring" will be available to the user. This drop down menu allows the user to either select the "Create Optimisation Set" or the "Automation" option on the menu. These two features have slightly different functions.

Create optimisation set

The "Create Optimisation Set" tab allows the user to create a non-automated optimisation set by manually inputting each new set of reaction conditions into the LC software. Upon selecting the "Create Optimisation Set" tab any data previously stored in the Chemstation registers is refreshed, and the user is presented with an input dialog box. This dialog box allows the user to define the reactor volume, the data set save location, the template method to be used, and the location of the input .txt data file. The user can then select the start button which generates the new reaction conditions (based on the

reaction conditions outlined within the .txt file previously generated). This method is added to a sequence which defines each of the individual reaction conditions which have been or will be run within the new optimisation set. Once the new set of conditions have been run, the user can manually analyse the data generated and determine the next set of conditions they wish to run in the optimisation sequence.

Automation

The "Automation" tab allows the user to create a fully automated optimisation set, whereby the Chemstation and visual macros control the entire optimisation. Again, having selected the "Automation" tab any data previously stored in the Chemstation registers is refreshed, and the user is presented with an input dialog box. This dialog box allows the user to define the reactor volume, the data set save location, the template method to be used, and the location of the input .txt data file. This time however, when the user selects the start tab the software takes over complete control of the optimisation, which will continue to run until pre-defined stopping criteria are reached. Selection of this tab loads a second macro (AC_S_Main) which opens and reads the data within the .txt file. If there is no text file available, the macro will wait until one appears. A third macro (AC_S_CreateMethod) is loaded and the .txt file is deleted. The CreateMethod macro defines the new LC method settings including the reaction flow rate, reaction temperature and reagent composition. The new method is then saved with a unique name corresponding to its individual experiment number. The macro will then calculate the reaction residence time from the new flow rate and the previously inputted reactor volume. This residence time is inputted into the method as a "post-time" delay, which gives the new reaction conditions time to equilibrate before a slug of the flow is injected onto the column. The new method conditions are saved and a fourth macro (AC_AddToSequence) is loaded. The add sequence macro creates a new row within the current sequence and adds the existing method to that row. This sequence is saved and once the equilibration delay period has been completed the new method will be run. Once the LC analysis for the run has been completed, a post run macro (AC_S_FindProduct) is loaded. Various iterations of this macro were produced, altering the data outputted within the .txt file. These iterations included data output as reaction yield, peak intensity, and peak area. This data is then output as another .txt file, which will be read either manually or by an external piece of optimisation software. The method then reloads AC_Main and the process repeats itself. This loop will continue until no more .txt files are available to be read.

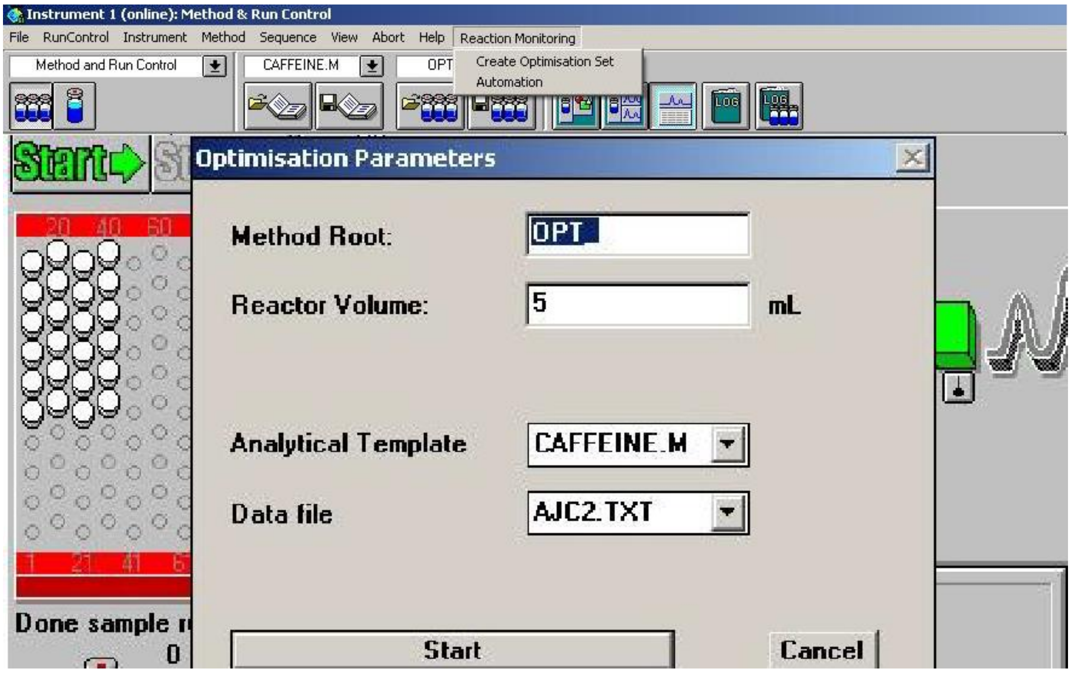

Figure S1: Example of the optimisation graphical user interface in Chemstation.

2.2 MacroPad macros

MacroPad is a piece of macro development software specifically designed for developing macros for the Chemstation software. Through MacroPad, it is possible to access the Chemstation registers, which store all the input and output variables produced during the HPLC analysis. The information stored in these registers allows control over variables such as reaction flow rates, temperature and pressure, as well as quantitative outputs such as spectroscopic and chromatographic data from any HPLC analysis undertaken. Using this software, it was possible to define the specific reaction and analysis conditions for each optimisation that was undertaken. The control and command of the HPLC software would need to be coupled with a separate piece of software which could handle the data generated by the flow system, as well as run a SIMPLEX type algorithm which would allow the output of a new set of reactions conditions back to the flow system. The optimisation software was designed so that it would recognise an external data file generated by the Chemstation software, containing the quantitative analysis data from the previous reaction run. This data would then be inputted into a spreadsheet, allowing the software to generate a new set of reaction conditions based upon the relative success of the previous reaction. These new reaction conditions would then be outputted as a data file, which would be read by the Chemstation software, generating a new set of reaction conditions. This optimisation loop would continue until a pre-defined set of criteria had been satisfied.

3. Experimental

3.1 General procedure for the formation of carvone semicarbazide

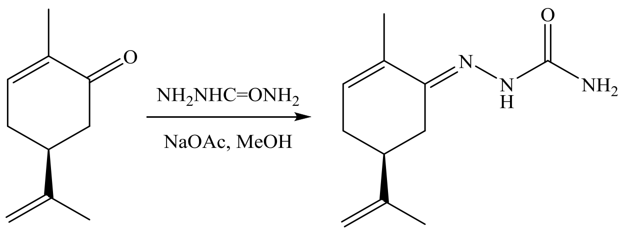

For specific reagent concentrations, flow regimes and analysis methods see results and discussion section of the main paper. *R*-(-) carvone was dissolved in methanol and transferred to a sample vial. To a second sample vial a solution containing semicarbazide hydrochloride, sodium acetate and water was prepared. The two solutions were mixed via a T-piece, and pumped at flow rates ranging from 0.1–1 mL/min through a number of different flow devices. The carvone semicarbazone product was analysed online via HPLC as well as in line via UV–vis spectroscopy.

3.2 General procedure for the formation of the fused polycyclic heterocylcle

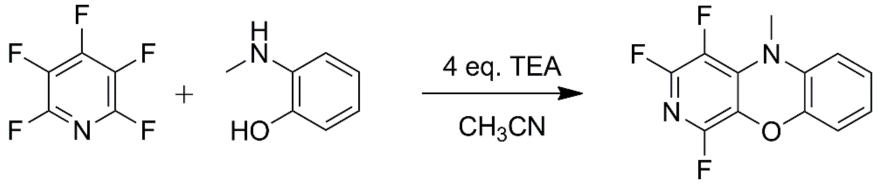

For specific reagent concentrations, flow regimes and analysis methods see results and discussion section of the main paper. Pentafluoropyridine was dissolved in acetonitrile and transferred to a sample vial. To a second sample vial a solution containing 2-(methylamino)phenol, trimethylamine and acetonitrile was prepared. The two solutions were mixed via a T-piece, and pumped at flow rates ranging from 0.1–-1 mL/min through a number of different flow devices. The heterocyclic product was analysed online via UV–vis absorption spectroscopy.

white solid. m.p. 150-152 °C.

NMR δ_H (400 MHz, $CDCl_3$), 6.94 (1H, td, *J* =8.0, 1.6 Hz, C-7), 6.85 (1H, td, *J* =8.0, 1.6 Hz, C-8), 6.77 (1H, dd, *J* =8.0, 1.6 Hz, C-9), 6.67 (1H, *J* =7.6, 1.2 Hz, C-6), 3.37 (3H, d, *J* =4.4 Hz).

NMR δ_F (376 MHz, $CDCl_3$), 68.8 (1F, dd, *J* =22, 15 Hz, F-1), 66.7 (1F, dd, *J* =22, 15 Hz, F-3), -0.2 (1F, tq, *J*=22, 5 Hz, F-4).

NMR δ_C (100 MHz, $CDCl_3$), 145.6(dt, *J* =235, 17 Hz, C-3), **145.3**, 142.9 (dd, *J* =235, 15 Hz, C-1), 136.5 (dt, *J* = 6, 2 Hz, C-4a), **132.4**, 130.6 (dd, *J* =247, 6 Hz, C-4), 126.39 (dd, *J* =28, 5 Hz, C-1a), 124.9 (C-7), 124.1 (C-8), 116.2 (C-9), 113.5 (C-6), 35.6 (d, *J* =12.4 Hz, CH_3).

IR, ν_{max}/cm^{-1} 1643 and 1574 (benzene ring)

MS, m/z found 253.0581, $C_{12}H_8F_3N_2O$, $(M+H^+)$ requires 253.0594.

3.3 Experimental details for RD1

For this optimisation an Agilent 1100 series binary pumping module was used to pump the two reagent flows, which passed through a 5 mL stainless steel coil reactor. This reactor was attached to a heating mandrel, and heated using the temperature controlled heating module of a Uniqsis FlowSyn. The flow would then pass into a six port valve, allowing it to be redirected into either a collection vial, or pass through RD1 for spectroscopic data collection (Figure S2).

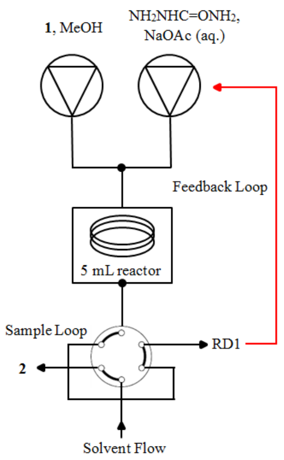

Figure S2: Reactor setup for carvone optimisation using RD1 as an inline spectroscopic flow cell. Reagents were pumped using an Agilent 1100 series HPLC pumping module. A Uniqsis FlowSyn was used to heat and cool the 5 mL stainless steel coil reactor. The flow passed onto a stand-alone six port valve, whereby samples were either passed into a collection vial or passed through RD1 which sat within the DAD compartment of the same Agilent 1100 series HPLC.

Table S3: Conditions and limits for the optimisation used in tandem with RD1. Ketone concentration 0.40 mmol/L, semicarbazide concentration 1.20 mmol/L.

Optimisation variable	Value
**Flow rate range**	0.2–1 mL/min
**Temperature range**	25–80 °C
**SIMPLEX temperature variation**	5 °C
**SIMPLEX flow rate variation**	0.1 mL/min
**Maximum data points**	30

The analysis macro used during this specific optimisation would monitor the intensity of absorption at a single predetermined wavelength (275 nm). At this wavelength the carvone starting material does not absorb the light, whereas the semicarbazone product does. The increase in intensity of absorbance at this value could therefore be attributed to the presence of an increased concentration of the reaction product. Prior to each new set of experimental conditions, the flow cell would be flushed with a MeOH /water mix (1:1 ratio), allowing the detector to be re-balanced.

3.4 Experimental details for RD2

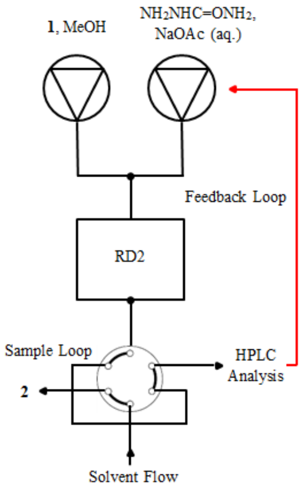

Figure S3: Reactor setup for carvone optimisation using RD2 in combination with online HPLC analysis. Reagents were pumped using an Agilent 1100 series HPLC pumping module. The temperature controlled HPLC column compartment was used to heat and cool RD2. The flow passed into a stand-alone six port valve, whereby samples were either passed into a collection vial or passed onto the HPLC column for online analysis of the reaction medium.

Table S4: Conditions and limits for the optimisation used in tandem with RD2. Ketone concentration 10 mmol/L, semicarbazide concentration 30 mmol/L.

**Optimisation variable**	**Value**
**Flow rate range**	0.2–1 mL/min
**Temperature range**	25–80 °C
**SIMPLEX temperature variation**	5 °C
**SIMPLEX flow rate variation**	0.1 mL/min
**Maximum data points**	40

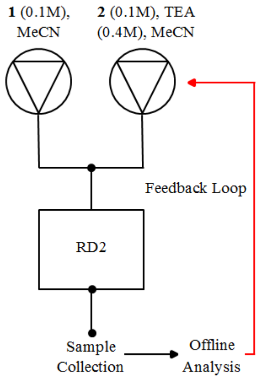

Figure S4: Reactor setup for the optimisation of the heterocyclic product using RD2 in combination with UV–vis spectroscopy. Reagents were pumped and heated using a FlowSyn system and analysed using an Ocean-Optics spectrophotometer.

Table S5: Conditions and limits for the optimisation used in tandem with RD2. Pentafluoropyridine concentration 0.1 mol/L, semicarbazide concentration 0.1 mol/L, trimethylamine concentration 0.4 mol/L.

**Optimisation variable**	**Value**
**Flow rate range**	0.4–1.2 mL/min
**Temperature range**	100–180 °C
**SIMPLEX temperature variation**	7 °C
**SIMPLEX flow rate variation**	0.16 mL/min
**Maximum data points**	20

3.5 Experimental details for RD3

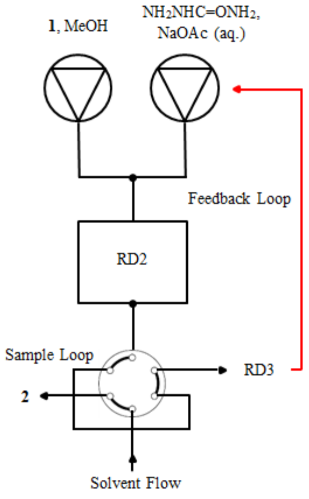

Figure S5: Reactor setup for carvone optimisation using RD2 in combination with inline spectroscopic UV–vis analysis via RD3. Reagents were pumped using an Agilent 1100 series HPLC pumping module RD3, whilst the temperature was controlled using the HPLC column compartment. The flow passed into a stand-alone six port valve, whereby samples were either passed into a collection vial or passed through RD3 which sat within the DAD compartment of the same Agilent 1100 series HPLC.

Table S6: Conditions and limits for the optimisation used in tandem with RD2 and RD3. Ketone **1** concentration 0.4 mmol/L, semicarbazide concentration 1.2 mmol/L.

**Optimisation variable**	**Value**
**Flow rate range**	0.2 – 1 mL/min
**Temperature range**	25 – 80 °C
**SIMPLEX temperature variation**	5 °C
**SIMPLEX flow rate variation**	0.1 mL/min
**Maximum data points**	20

3.6 Reactor temperature verification

To assess the temperature variation between the set temperature of the Agilent Column Compartment and the actual temperature of the solvent, we measured the solvent temperature against the set point. Figure S6 shows the difference between these. There is an offset of around 5 °C per increase of 20 °C. There is a small difference between the flow rates.

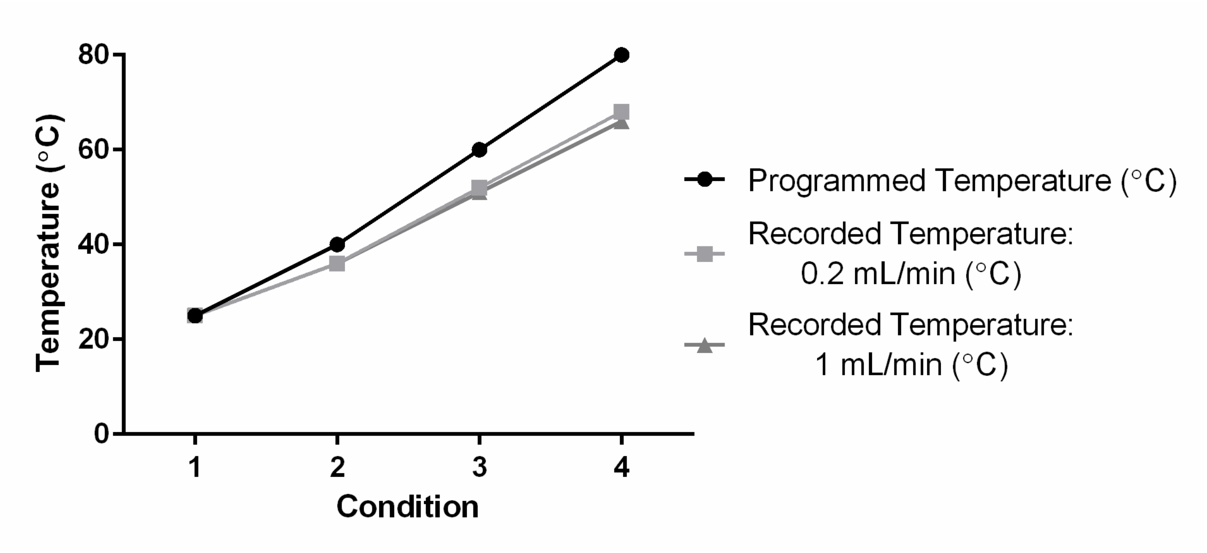

Figure S6: Temperature variation.
